# Supplementary material for: Heat adaptation of phage T7 under an extended genetic code
Source: Virus Evol. 2021 Dec 1;7(2):veab100. doi: 10.1093/ve/veab100 (PMC8923235; doi:10.1093/ve/veab100)
Supplement: veab100_Supp [file veab100_supp.zip › Sequencing_Analysis.zip!/Sequencing_Analysis/A3/evidence/RA_30.html]

BRESEQ :: Evidence


| Read alignment evidence... | | | | | | | | | | | |
| --- | --- | --- | --- | --- | --- | --- | --- | --- | --- | --- | --- |
|  | seq id | position | | ref | new | freq | score (cons/poly) | reads | annotation | genes | product |
| \* | NC\_001604.1 | 19,625 | 0 | G | T | 20.0% | 3611.2 / inf | 1523 | intergenic (–/–) | *–/–* | –/– |
| Reads supporting (aligned to +/- strand):  **ref** base G (576/637);  **new** base T (157/147);  **total** (737/786) | | | | | | | | | | | |
| Fisher's exact test for biased strand distribution *p*-value = 2.00e-01 | | | | | | | | | | | |
| Kolmogorov-Smirnov test that lower quality scores support variant *p*-value = 1.00e+00 | | | | | | | | | | | |

Only 100 of 1540 total aligned reads displayed.  

`GCGTGGTGCGTAAGCTGTGACATTCACCACAAAGGAGACATTCTGGATGTAACGTCCGTTGTCATTAATCCTGAGGCAGACTCTAAGGGCTTACAGCGATTCCTAGCGAAACGCTTTAAGTACCTTGCGGAACTCCACGATTGCGATTGGGTGTCTCGTTGTAAGCATGAAGGCGAGACAATGCGTGTATACTTTAAGGAGGTATAAGTTATGGGTAAGAAAGTTAAGAAGGCCGTGAAGAAAGTCACCAAGTCCGTTAAGAAAGTCGTTAAGGAAGGGGCTCGTCCGGTTAAACAGGTTGCTGGCGGTCTAGCTGGTCTGGCTGGTGGTACTGGTGAAGCACAGATGGTGGAAGTACCACAAGCTGCCGCACAGATTGTTGACGTACCTGAGAAAGAGGTTTCCACTGAGGACGAAGCACAGACAGAAAGCGGACGCAAGAAAGCTCGTGCTGGCGGTAAGAAATCCTTGAGTGTAGCCCGTAGCTCCGGTGGCGGTATCAACATTTAATCAGGAGGTTATCGTGGAAGACTGCATTGAATGGACCGGAGGTGTCAACTCTAAGGGTTATGGTCGTAAGTGGGTTAATGGTAAACT  >  NC_001604.1/19325‑19921`  

                                                                                                                                                                                                                                                                                                            `|`                                                                                                                                                                                                                                                                                                          
     `gtgCGTAAGCTGTGACATTCACCACAAAGGAGACATTCTGGATGTAACGTCCGTTGTCATTAATCCTGAGGCAGACTCTAAGGGCTTACAGCGATTCCTATCGAAACGCTTTAAGTACCTTGCGGACCTCCACGATTGCGATTGGGTGTCTCGTTGTAAGCATGAAGGCGAGACAATGCGTGTATACTTTAAGGAGGTATAAGTTATGGTTAAGAAAGTTAAGAAGGCCGTGAAGAAATTCACCAAGTCCGTTAAGAAAGTCGTTAAGGAAGGGGCTCGTCCGGTTAAACAGGTTGCTGGc                                                                                                                                                                                                                                                                                                     >  1:71123/1‑301 (MQ=255)`  
     `gtgCGTAAGCTGTGACATTCACCACAAAGGAGACATTCTGGATGTAACGTCCGTTGTCATTAATCCTGAGGCAGACTCTAAGGGCTTACAGCGATTCCTAGCGAAACGCTTTAAGTACCTTGCTGAACTCCTCGATTGCGATTGGGTGTCTCGTTGTAAGCATGAAGGCGAGACAATGCGTGTATACTTTAAGGAGGTATAAGTTATGGGTAAGAAAGTTAAGAAGGCCGTGAAGAAAGTCACCAAGTCCGTTAAGAAAGTCGTTAAGGAAGGGGCTCGTCCGGTTAAACAGGTTGCTggg                                                                                                                                                                                                                                                                                                     >  1:181219/1‑300 (MQ=255)`  
                `gagatgtgtataagagacaggaCATTCTGGATGTAACGTCCGTTGTCATTAATCCTGAGGCAGACTCTAAGGGCTTACAGCGATTCCTAGCGAAACGCTTTAAGTACCTTGCGGAACTCCACGATTGCGATTGGGTGTCTCGTTTTAAGCATGAAGGCGAGGCAATGCGTGTATACTTTAAGGAGGTATAAGTTATGGGTAAGAAAGTTAAGAAGGCCGTGAAGAAAGACACCAAGTCCGTTAAGAAAGTCGTTAAGGAAGGGGCTCGTCCGGTTAAACAGGTTTCTGGCGGTCTAGCTgg                                                                                                                                                                                                                                                                                          <  1:15152/281‑1 (MQ=255)`  
                   `aCATTCACCACAAAGGAGACATTCTGGATGTAACGTCCGTTGTCATTAATCCTGAGGCAGACTCTAAGGGCTTACAGCGATTCCTAGCGAAACGCTTTAAGTACCTTGCGGAACTCCACGATTGCGATTGGGTGTCTCGTTGTAAGCATGAAGGCGAGACAATGCGTGTATACTTTAAGGAGGTATAAGTTATGGGTAAGAAAGTTAAGAAGGCCGTGAAGAAAGTCACCAAGTCCGTTAAGAAAGTCGTTAAGGAAGGGGCTCGTCCGGTTAAACAGGTTGCTGGCGGTCTggctggtgg                                                                                                                                                                                                                                                                                       >  1:271356‑M1/1‑292 (MQ=255)`  
                     `aTTCACCACAAAGGAGACATTCTGGATGTAACGTCCGTTGTCATTAATCCTGAGGCAGACTCTAAGGGCTTACAGTGATTCCTAGCGAAACGCTTTAAGTACCTTGCGGAACTCCACGATTGCGATTGGGTGTCTCGTTGTAAGCATGAAGGCGAGACAATGCGTGTATACTTTAAGGAGGTATAAGTTATGGGTAAGAAAGTTAAGAAGGCCGTGAAGAAAGTCACCAAGTCCGTTAAGAAAGTCGTTAAGGAAGGGGCTCGTCCGGTTAAACAGGTTTCTGGCGGTCTAGCTGGTctgg                                                                                                                                                                                                                                                                                     >  1:334600/1‑301 (MQ=255)`  
                     `aTTCACCACAAAGGAGACATTCTGGATGTAACGTCCGTTGTCATTAATCCTGAGGCAGACTCTAAGGGCTTACAGCGATTCCTAGCGAAACGCTTTAAGTACCTTGCGGAACTCCACGATTGCGATTGGGTGTCTCGTTGTAAGCATGAAGGCGAGACAATGCGTGTATACTTTAAGGAGGTATAAGTTATGGGTAAGAAAGTTAAGAAGTCCGTGAAGAAAGTCACCAAGTCCGTTAAGAAAGTCGTTAAGGAAGGGGCTCGTCCGGTTAAACAGGTTGCTGGCGGTCTggctggtggta                                                                                                                                                                                                                                                                                     >  1:255170‑M1/1‑290 (MQ=255)`  
                          `ccacAAAGGAGACATTCTGGATGTAACGTCCGTTGTCATTAATCCTGAGGCAGACTCTAAGGGCTTACAGCGATTCCTAGCGAAACGCTTTAAGTACCTTGCGGACCTCCACTATTGCGATTGGGTGTCTCGTTGTAAGCATGAAGGCGAGACAATGCGTGTATACTTTAAGGAGGTATAAGTTATGGGTAAGAAAGTTAAGAAGGCCGTGAAGAAAGTCACCAAGTCCGTTAAGAAAGTCGTTAAGGAAGGGGCTCGTCCGGTTAAACAGGTTGCTGGCGGTCTgtctggtggtactggt                                                                                                                                                                                                                                                                                >  1:173141‑M1/1‑285 (MQ=255)`  
                           `atgtgtataagagacagggatggaacgtccgttgtcATTAATCCTGAGGCAGACTCTAAGGGCTTACAGCGATTCCTAGCGAAACGCTTTAAGTACCTTGCGGAACGCCACGATTGCGATTGGGTGTCTCGTTGTAAGCATGAAGGCGAGACAATGCGTGTATACTTTAAGGAGGTATAAGTTATGGGTAAGAAAGTTAAGAAGGCCGTGAAGAAAGTCACCAAGTCCGTTAAGAAAGTCGTTAAGGAAGGGGCTCGTCCGGTTAAACAGGTTGCTGGCGGTCTggctggtggtactggtg                                                                                                                                                                                                                                                                               <  1:153468‑M1/284‑18 (MQ=255)`  
                             `caAAGGAGACATTCTGGATGTAACGTCCGTTGTCATTAATCCTGAGGCAGACTCTAAGGGCTTACAGCGATTCCTAGCGAAACGCTTTAAGTACCTTGCGGAACTCCACGATTGCGATTGGGTGTCTCGTTGTAAGCATGAAGGCGAGACAATGCGTGTATCCTTTAAGGAGGTATAAGTTATGGGTAAGAAAGTTAAGAAGGCCGTGAATAAATTCACCAAGTCAGTTAATAAAGTCGTTAAGGAAGGGGCTCGTCCGGTTAAACAGGTTGCTGGCGGTCTggctggtggtactggtgaa                                                                                                                                                                                                                                                                             >  1:22941‑M1/1‑282 (MQ=255)`  
                                 `ggAGACATTCTGGATGTAACGTCCGTTGTCATTAATCCTGAGGCAGACTCTAAGGGCTTACAGCGATTCCTAGCGAAACGCTTTAAGTACCTTGCGGAACTCCACGATTGCGATTGGGTGTCTCGTTGTAAGCATGAAGGCGAGACAATGCGTGTATACTTTAAGGAGGTATAAGTTATGGGTAAGAAAGTTAAGAAGGCCGTGAAGAAAGTCACCAAGTCCGTTAAGAAAGTCGTTAAGGAAGGGGCTCGTCCGGTTAAGCAGGTTGCTGGCGGTCTggctggtggtcctggtgaagcac                                                                                                                                                                                                                                                                         >  1:326113‑M1/1‑278 (MQ=255)`  
                                 `ggAGACATTCTGGATGTAACGTCAGTTGTCATTAATCCTGAGGCAGACTCTAAGGGCTTACAGCGATTCCTAGCGAAACGCTTTAAGTACCTTGCGGAACTCCACGATTGCGATTGGGTGTCTCGTTGTAAGCATGAAGGCGAGACAATGCGTGTATACTTTAAGGAGGTATAAGTTATGGGTAAGAAAGTTAAGAAGGCCGTGAAGAAAGTCACCAAGTCCGTTAAGAAAGTCGTTAAGGAAGGGGCTCGTCCGGTTAAACAGGTTGCTGGCGGTCTggctggtggtactggtgaagcac                                                                                                                                                                                                                                                                         >  1:205410‑M1/1‑278 (MQ=255)`  
                                     `aCATTCTGAATGTAATGTCCGTTTTCATTAATCCTGAGGCAGACTCTAAGGGCTTACAGCGATTCCTAGCGAAACGCTTTAAGTACCTTGCGGAACTCCACGATGGCGATTGGGTGTCTCGTTGTAAGCATGAAGGCGAGACAATGCGTGTATACTTTAAGGAGGTATAAGTTATGGGTAAGAAAGTTAAGAAGGCCGTGAAGAAAGTCACCAAGTCCGTTAAGAAAGTCGTTAAGGAAGGGGCTCGTCCGGTTAAACAGGTTGCTGGCGGTCTggctggtggtactggtgaagcacagat                                                                                                                                                                                                                                                                     <  1:2005‑M1/301‑28 (MQ=255)`  
                                        `ttCTGGATGTAACGTCCGTTGTCATTAATCCTGAGGCAGACTCTAAGGGCTTACAGCGATTCCTAGCGAAACGCTTTAAGTACCTTGCGGAACTCCACGATTGCGATTGGGTGTCTCGTTGTAAGCATGAAGGCGAGACAATGCGTGTATACTTTAAGGAGGTATAAGTTATGGGTAAGAAAGTTAAGAAGGCCGTGAAGAAAGTCACCAAGTCCGTTAAGAAAGTCGTTAAGGAAGGGGCTCGTCCGGTTAAACAGGTTGCTGGCGGTCTggctggtggtactggtgaagcacagatgct                                                                                                                                                                                                                                                                  >  1:88211‑M1/1‑271 (MQ=255)`  
                                         `ggagatgtgtataagagacagcattaatcctgaggcagactctAAGGGCTTACAGCGATTCCTAGCGAAACGCTTTAAGTACCTTGCGGAACTCCACGATTGCGATTGGGTGTCTCGTTGTAAGCATGAAGGCGAGACAATGCGTGTATACTTTAAGGAGGTAGAAGTTATGGGTAAGAAAGTTAAGAAGGCCGTGAAGAAAGTCACCAAGTCCGTTAAGAAAGTCGTTAAGGAAGGGGCTCGTCCGGTTAAACAGGTTGCTGGAGGTCTggctggtggtactggtgaagcacagatggtg                                                                                                                                                                                                                                                                 <  1:143609‑M1/280‑32 (MQ=255)`  
                                           `gctcggagatgtgtataagagacagtcctgaggcagactctaagggcttacAGCGATTCCTAGCGAAACGCTTTAAGTACCTTGCGGAACTCCACGATTGCGATTGGGTGTCTCGTTGTAAGCATGAAGGCGAGACAATGCGTGTATACTTTAAGGAGGTATAAGTTATGGGTAAGAAAGTTAAGAAGGCCGTGAAGAAAGTCACAAAGTCCGTTAAGAAAGTCGTTAAGGAAGGGGCTCGTCCGGTTAAACAGGTTGCTGGCGGTCTggctggtggtactggtgaagcacagatggtgga                                                                                                                                                                                                                                                               <  1:30586‑M1/276‑34 (MQ=255)`  
                                            `ggATGTAACGTCCGTTGTCATTAATCCTGAGGCAGACTCTAAGGGCTTACAGCGATTCCTAGCGAAACGCTTTAAGTACATTGCGGAACTCCACGATTGCGATTGGGTGTCTCGTTGTAAGCATGAAGGCGAGACAATGCGTGTATACTTTAAGGAGGTATAAGTTATGGGTAAGAAAGTTAAGAAGGCCGTGAAGAAAGTCACCAAGTCCGTTAAGAAAGTCGTTAAGGAAGGGGCTCGTCCGGTTAAACAGGTTGCTGGCGGTCTggctggtggtactggtgaaggacagatggtggaa                                                                                                                                                                                                                                                              >  1:39008‑M1/1‑267 (MQ=255)`  
                                              `aTGTAACGTCCGTTGTCATTAATCCTGAGGCAGACTCTAAGGGCTTACAGCGATTCCTAGCGAAACGCTTTAAGTACCTTGCGGAACTCCACGATTGCGATTGGGTGTCTCGTTGTAAGCATGAAGGCGAGACAATGCGTGTATACTTTAAGGAGGTATAAGTTATGGGTAAGAAAGTTAAGAAGGCCGTGAAGAAAGTCACCAAGTCCGTTAAGAAAGTCGTTAAGGAAGGGGCTCGTCCGGTTAAACAGGTTGCTGGCGGTCTggctggtggtactggtgaagcacggatggtggaagt                                                                                                                                                                                                                                                            >  1:315131‑M1/1‑265 (MQ=255)`  
                                                    `cGTCCGTTGTCATTAATCCTGAGGCAGACTCTAAGGGCTTACAGCGATTCCTAGCGAAACGCTTTAAGTACCTTGCGGAACTCCACGATTGCGATTGGGTGTCTCGTTGTAAGCATGAAGGCGAGACAATGCGTGTATACTTTAAGGAGGTATAAGTTATGGGTAAGAAAGTTAAGAAGGCCGTGAAGAAAGTCACCAAGTCCGTTAAGAAAGTCGTTAAGGAAGGGGCTCGTCCGGTTAAACAGGTTTCTGGCGGTCTAGCTGGTCTGGCTGGTGGTACTGGTGAAGCACAGATGGTGGa                                                                                                                                                                                                                                                      >  1:224603/1‑301 (MQ=255)`  
                                                    `cGTCCGTTGTCATTAATCCTGAGGCAGACTCTAAGGGCTTACAGCGATTCCTAGCGAAACGCTTTAAGTACCTTGCGGAACTCCACGATTGCGATTGGGTGTCTCGTTGTAAGCATGAAGGCGAGACAATGCGTGTATACTTTAAGGAGGTATAAGTTATGGGTAAGAAAGTTAAGAAGGCCGTGAAGAAAGTCACCAAGTCCGTTAAGAAAGTCGTTAAGGAAGGGGCTCGTCCGGTTAAACAGGTTGCTGGCGGTCTggctggtggtactggtgaagcacagatggtggaagtaccaca                                                                                                                                                                                                                                                      >  1:131370‑M1/1‑259 (MQ=255)`  
                                                      `caccatttCATTAAGCCTGGAGCAGACTGAAAGGACTTACAGCGATCCCGAGCGAAACGCTTGAAGTAACTTGCGGAACTCCACGATTGCGATGGTGTGTGTCGTTGTAAGAATGAAGGCGAGACAATGTGAGTATACTTTAAGGAGGAATAAGTGATGGGTAAGAAAGTGAAGAAGGCCGAGAAGAAAGTCACCAAGTCCGTTAAGAAAGTCGTTAAGGAAGGGGCTCGTCCGGTTAGACAGGTTGCGGGCGGTCTAGCTGGTCTGGCTGGTGGTACTGGTGAAGCACAGATGGTGGAAg                                                                                                                                                                                                                                                    <  1:10347/294‑1 (MQ=255)`  
                                                            `gTCATTAATCCTGAGGCAGACTCTAAGGGCTTACAGCGATTCCTAGCGAAACGCTTTAAGTACCTTGCGGAACTCCACGATTGCGATTGGGTGTCTCGTTGTAAGCATGAAGGCGAGACAATGCGTGTATACTTTAAGGAGGTATAAGTTATGGGTAAGAAAGTTAAGAAGGACGTGAAGAAAGTCACAAAGTCCGTTAAGAAAGTCGTTAAGGAAGGGGCTCGTCCGGTTAAACAGGTTTCTGGCGGTCTAGCTGGTCTGGCTGGTGGTACTGGTGAAGCCCAGATGGTGGAAGTACcac                                                                                                                                                                                                                                              >  1:278065/1‑301 (MQ=255)`  
                                                              `cATTAATCCTGAGGCAGACTCTAAGGGCTTACAGCGATTCCTAGCGAAACGCTTTAAGTACCTTGCGGAACTCCACGATTGCGATTGGGTGTCTCGTTGTAAGCATGAAGGCGAGACAATGCGTGTATACTTTAAGGAGGTATAAGTTATGGGTAAGAAAGTTAAGAAGGCCGTGAAGAAAGTCAGCAAGTCCGTTAAGAAAGTCGTTAAGGAAGGGGCTCGTCCGGTTAAACAGGTTGCTGGCGGTCTggctggtgatactggtgaagcacagatggtggaagtaccacaagctgcagca                                                                                                                                                                                                                                            >  1:129130‑M1/1‑249 (MQ=255)`  
                                                               `ggctcggagatgtgtttaagagacagcttacagcgatccctggcgaaacgcTTTAAGTACCTTGCGGAACTCCACGATTGCGATTGGGTGTCTCGTTGTAAGCATGAAGGCGAGACAATGCGTGTATACTTTAAGGAGGTATAAGTTATGGGTAAGAAAGTTAAGAAGGCCGTGAAGAAAGTCACCAAGTCCGTTAAGAAAGTCGTTAAGGAAGGGGCTCGTCCGGTTAAACAGGTTGCTGGCGGTCTggctggtggtactggtgaagcacagatggtggaagtaccacaagctgccgcac                                                                                                                                                                                                                                           <  1:226487‑M1/276‑54 (MQ=255)`  
                                                               `agtaaTCCGGAGGCAGACTCTAAGGGCTTACAGCGATTCCTAGCGAAACGCTTTAAGTACCTTGCGGAACTCCACGATTGCGATTGGGTGTCTCGTTGTAAGCATGAAGGCGAGACAATGCGTGTATACTTTAAGGAGGTATAAGTTATGGGTAAGAAAGTTAAGAAGGCCGTGAAGAAAGTCACCAAGTCCGTTAAGAAAGTCGTTAAGGAAGGGGCTCGTCCGGTTAAACAGGTTGCTTGCGGTCTggctggtggtactggtgaagcacagatggtggaaggaccacaagctgccgcac                                                                                                                                                                                                                                           <  1:119462‑M1/299‑54 (MQ=255)`  
                                                                    `tCCTGAGGCAGACTCTAAGGGCTTACAGCGATTCCTAGCGAAACGCTTTAAGTACCTTGCGGAACTCCACGATTGCGATTGGGTGTCTCGTTGTAAGCATGAAGGCGAGACAATGCGTGTATACTTTAAGGAGGTATAAGTTATGGGTAAGAAAGTTAAGAAGGCCGTGAAGAAAGTCACCAAGTCCGTTAAGAAAGTCGTTAAGGAAGGGGCTCGTCCGGTTAAACAGGTTTCTGGCGGTCTAGCTGGTCTGGCTGGTGGTACTGGTGAAGCACAGATGGTGGAAGTACCACAAGCTGcc                                                                                                                                                                                                                                      >  1:51797/1‑301 (MQ=255)`  
                                                                        `gAGGCAGACTCTAAGGGCTTACAGCGATTCCTAGCGAAACGCTTTAAGTACCTTGCGGAACTCCACGATTGCTATTGGGTGTCTCGTTGTAAGCATGAAGGCGAGACAATGCGTGTATACTTTAAGGAGGTATAAGTTATGGGTAAGAAAGTTAAGAAGGCCGTGAAGAAAGTCACCAAGTCCGTTAAGAAAGTCGTTAAGGAAGGGGCTCGTCCGGTTAAACAGGTTGCTGGCGGTCTggctggtggtactggtgaagcacagatggtggaagtacaacaagctgccgcacagatggttg                                                                                                                                                                                                                                  >  1:163838‑M1/1‑239 (MQ=255)`  
                                                                                `ctctAAGGGCTTACATCGATTCCTAGCGAAACGCTTTAAGTACCTTGCGGAACTCCACGATTGCGATTGGGTGTCTCGTTGTAAGCATGAAGGCGAGACAATGCGTGTATACTTTAAGTAGGTATAAGTTATGGGTAAGAAAGTTAAGAAGGCCGTGAAGAAAGTCACCAAGTCCGTTAAGAAAGTCGTTAAGGAAGGGGCTCGTCCGGTTAAACAGGTTGCTGGCGGTCTggctggtggtactggtgaagcacagatggtggaagtaccacaagctgccgcacagattgttgacgctgtc                                                                                                                                                                                                                          >  1:93777‑M1/1‑231 (MQ=255)`  
                                                                                  `ctAAGGGCTTACCGCGATTCCTAGCGAAACGCTTTAAGTACCTTGCGGAACTCCACGATTGCGATTGGGTGTCTCGTTGTAAGCATGAAGGCGAGACAATGCGTGTATACTTTAAGGAGGTATAAGTTATGGGTAAGAAAGTTAAGAAGGCCGTGAAGAAAGTCACCAAGTCCGTTAAGAAAGTCGTTAAGGAAGGGGCTCGTCCGGTTAAACAGGTTTCTGGCGGTCTAGCTGGTATGGCTGGTGGTACTGGTGAAGCACAGATGGTGGAAGTACCACAAGCTGCCGCACAGATTGTTGa                                                                                                                                                                                                                        <  1:970/301‑1 (MQ=255)`  
                                                                                       `ggCTTACAGCGATTCCTAGCGAAACGCTTTAAGTACCTTGCGGAACTCCACGATTGCGATTGGGTGTCTCGTTGTAAGCATGAAGGCGAGACAATGCGTGTATACTTTAAGGAGGTATAAGTTATGGGTAAGAAAGTTAAGAAGGCCGTGAAGAAAGTCACCAAGTCCGTTAAGAAAGTCGTTAAGGAAGGGGCTCGTCCGGTTAAACAGGTTTCTGGCGGTCTAGCTGGTCTGGCTGGTGGTACTGGTGAAGCACAGATGGTGGAAGTACCACAAGCTGCCGCACAGATTGTTGACGTAc                                                                                                                                                                                                                   >  1:260189/1‑301 (MQ=255)`  
                                                                                          `ttACAGCGATTCCTAGCGAAACGCTTTAAGGACCTTGCGGAACTCCACGATTGCGATTGGGTGTATCGTTGTAAGCATGAAGGCGAGACAATGCGTGTATACTTTAAGGAGGTATAAGTTATGGGTAAGAAAGTTAAGAAGGCCGTGAAGAAAGTCACCAAGTCCGTTAAGAAAGTCGTTAAGGAAGGGGCTCGTCCGGTTAAACAGGTTTCTGGCGGTCTAGCTGGTCTGGCTGGTGGTACTGGTGAAGCACAGATGGTGGAAGTACCACAAGCTGCCGCACAGATTGTTGACGTACCTg                                                                                                                                                                                                                <  1:79272/301‑1 (MQ=255)`  
                                                                                                   `ttCCTAGCGAAACGCTTTAAGTACCTTGCGGAACTCCACGATTGCGATTGGGTGGCTCGTTGGAAGCATGAAGGCGAGAAAATGCGTGTAAACTTTAAGGAGGTATAAGTTATGGGTAAGAAAGTTAAGAAGGCCGGGAAGAAAGTCACCAAGGCCGTTAAGAAAGTCGTTAAGGAAGGGGCTCGTCCGGTTAAACAGGTTTCTGGCGGTCTAGCTGGTCTGGCTGGTGGTACTGGTGAAGCACAGATGGTGGAAGTACAACAAGCTGCCGCACAGATTGTTGACGTACCTGAGAAAGAgg                                                                                                                                                                                                       <  1:283089/301‑1 (MQ=255)`  
                                                                                                          `gagacagCTGTAAGTACCTTGCGGAACTCCACGATTGCGATGGGGTGTCTCGTTGTAAGCATGAAGGCGAGACAATGCGTGTATACTTTAAGGAGGTATAAGTTATGGGTAAGAAAGTTAAGAAGGCCATGAAGAAAGTCACCAAGTCCGTTAAGAAAGTAGTTAAGGAAGGGGCTCGTCCGGTTAAACAGGTTGCTGGCGGTCTAGCTGGTCTGGCTGGTGGTACTGGTGAAGCACAGATGGTGGAAGTACCACAAGCTGCCGCACAGATTGTTGACGTACCTGAGAAAGAGGTTTCCAc                                                                                                                                                                                                <  1:187123/295‑1 (MQ=255)`  
                                                                                                            `tgtttataggggacagttgcggaactccacgattGCGATTGGGTGTCTCGTTGTAAGCATGAAGGCGAGACAATGCGTGTATACTTTAAGGAGGTATAAGTTATGGGTAAGAAAGTTAAGAAGGCCGTGAAGAAAGTCACCAAGTCCGCTAAGAAAGTCGTTAAGGAAGGGGCTCGTCCGGTTAAACAGGTTGCTGGCGGTCTagctggtggtactggtgaagcacagatggtggaagtaccacaagctgccgcacagattgttgacgtacctgagaaagaggtttccactgaggacgaag                                                                                                                                                                                              <  1:13612‑M1/285‑99 (MQ=255)`  
                                                                                                              `aCGCTTTAAGTACCTTGCGGAACTCCACGATTGCGATTGGGTGTCCCGTTGTAAGCATGAAGGCGAGACAATGCGTGTATACTTTAAGGAGGTATAAGTTATGGGTAAGAAAGTTAAGAAGGCCGTGAAGAAAGTCACCAAGTCCGTTAAGAAAGTCGTTAAGGAAGGGGCTCGTCCGGTTAAACAGGTTGCTGGCGGTCTggctggtggtactggtgaagcacagatggtggaagtaccacaagctgccgcacagattgttgacgtacctgagaaagaggctgtctcttatacacatctc                                                                                                                                                                                            >  1:55072‑M1/1‑201 (MQ=255)`  
                                                                                                                 `cTTTAAGTACCTTGCGGAACTCCACGATTGCGATTGGGTGTCTCGTTGTAAGCATGAAGGCGAGACAATGCGTGTATACTTTAAGGAGGTATAAGTTATGGGTAAGAAAGTTAAGAAGGCCGTGAAGAAAGTCACCAAGTCCGTTAAGAAAGTCGTTAAGGAAGGGGCTCGTCCGGTTAAACAGGTTGCTGGCGGTCTggctggtggtactggtgaagcacagatggtggaagtaccacaagctgccgcacagattgttgacgtacctgagaaagaggtttccactgcggtctcttataca                                                                                                                                                                                         >  1:299782‑M1/1‑198 (MQ=255)`  
                                                                                                                  `tttAAGTACCTTGCGGAACTCCACGATTGCGATTGGTTGTCTCGTTGTAAGCATGAAGGCGAGACAATGCGTGTATACTTTAAGGAGGTATAAGTTATGGGTAAGAAAGTTAAGAAGGCCGTGAAGAAAGTCACCAAGTCCGTTAAGAAAGTCGTTAAGGAAGGGGCTCGTCCGGTTAAACAGGTTGCT‑‑‑GGTCTggctggtggtactggtgaagcacagatggtggaagtaccacaagctgccgcacagattgttgacgtacctgagaaagaggtttccactgaggacgaagcacagacag                                                                                                                                                                                     <  1:232563‑M1/301‑108 (MQ=255)`  
                                                                                                                   `ttAAGTACCTTGCGGAACTCCACGATTGCGATTGGGTGTCTCGTTGTAAGCATGAAGGCGAGACAATGCGTGTATACTTTAAGGAGGTATAAGTTATGGGTAAGAAAGTTAAGAAGGCCGTGAAGAAAGTCACCAAGTCCGTTAAGAAAGTCGTTAAGGAAGGGGCTCGTCCGGTTAAACAGGTTGCTGGCGGTCTggctggttgtactggtgaagcacagatggtggaagtaccacaagctgcctcacagattgttgacgtacctgagaaagatgtttccactgaggacgaagcacagac                                                                                                                                                                                       <  1:340276‑M1/301‑106 (MQ=255)`  
                                                                                                                   `ttAAGTACCTTGCGGAACTCCACGATTGCGATTGGGTGTCTCGTTGTAAGCATGAAGGCGAGACAATGCGTGTATACTTTAAGGAGGTATAAGTTATGGGTAAGAAAGTTAAGAAGGCCGTGAAGAAAGTCACCAAGTCCGTTAAGAAAGTCGTTAAGGAAGGGGCTCGTCCGGTTAAACAGGTTGCTGGCGGTCTggctggtggtactggtgaagcacagatggtggaagtaccacaagctgccgcacagattgttgacgtacctgagaaagaggtttccactgaggacgaagcacagac                                                                                                                                                                                       <  1:123206‑M1/301‑106 (MQ=255)`  
                                                                                                                   `ttAAGTACCTTGCGGAACTCCACGATTGCGATTGGGTGTCTCGTTGTAAGCATGAAGGCGAGACAATGCGTGTATACTTTAAGGAGGTATAAGTTATGGGTAAGAAAGTTAAGAAGGCCGTGAAGAAAGTCACCAAGTCAGTTAAGAAAGTCGTTAAGGAAGGGGCTCGTCCGGTTAAACAGGTTGCTGGCGGTCTggctggtggtactggtgaagcacagatggtggaagtaccacaagctgccgcacagattgttgacgtacctgagaaagaggtttccactgctgtctcttatacaca                                                                                                                                                                                       >  1:69231‑M1/1‑196 (MQ=255)`  
                                                                                                                   `agacagaccttgcGGAACTCCACGATTGCGATTGGGTGTCTCGTTGTAAGCATGAAGGCGAGACAATGCGTGTATACTTTAAGGAGGTATAAGTTATGGGTAAGAAAGTTAAGAAGGCCGTGAAGAAAGTCACCAAGTCCGTTAAGAAAGTCGTTAAGGAAGGGGCTCGTCCGGTTAAACAGGTTGCTGGCGGTCTggctggtggtactggtgaagcacagatggtggaagtaccacaagctgccgcacagattgttgacgtacctgagaaagaggtttccactgaggacgaagcacagac                                                                                                                                                                                       <  1:42238‑M1/295‑106 (MQ=255)`  
                                                                                                                     `aaGGACCTTGCGGAACTCCACGATTGCGATTGGGTGTCTCGTTGTAAGCATGAAGGCGAGACAATGCGTGTATACTTTAAGGAGGTATAAGTTATGGGTAAGAAAGTTAAGAAGGCCGTGAAGAAAGTCACCAAGTCCGTTAAGAAAGTCGTTAAGGAAGGGGCTCGTCCGGTTAAACAGGTTGCTGGCGGTCTggctggtggtactggtgaagcacagatggtggaagtaccacaagctgccgcacagattgttgacgtacctgagaaagaggtttcaactgaggacgaagcacagacag                                                                                                                                                                                     <  1:235945‑M1/301‑108 (MQ=255)`  
                                                                                                                         `gatgtgtataagagacagattgcgattgggtgtctcgttgtaagcATGAAGGCGAGACAATGCGTGTATACTTTAAGGAGGTATAAGTTATGGGTAAGAAAGTTAAGAAGGCCGTGAAGAAAGTCACCAAGTCCGTTAAGAAAGTCGTTAAGGAAGGGGCTCGTCCGGTTAAACAGGTTGCTGGCGGTCTggctggtggtactggtgaagcacagatggtggaagtaccacaagctgcagcacagattgttgacgtacctgagaaagaggtttccactgaggacgaagcacagacagaaag                                                                                                                                                                                 <  1:302678‑M1/284‑112 (MQ=255)`  
                                                                                                                         `aCCTTGCGGAACTCCACGATTGCGATTGGGTGTCTCGTTGTAAGCATGAAGGCGAGACAATGCGTGTATACTTTAAGGAGGTATAAGTTATGGGTAAGAAAGTTAAGAAGGCCGTGAAGAAAGTCACCAAGTCCGTTAAGAAAGTCGTTAAGGAAGGGGCTCGTCCGGTTAAACAGGTTGCTGGCGGTCTggctggtggtactggtgaagcacagatggtggaagtaccacaagctgccgcacagattgttgacgtacctgagaaagaggtttccactgaggacgaagcacagacagaaag                                                                                                                                                                                 >  1:169180‑M1/1‑190 (MQ=255)`  
                                                                                                                            `ttGCGGAACTCCACGATTGCGATTGGGTGTCTCGTTGTAAGCATGAAGGCGAGACAATGCGTGTATACTTTAAGGAGGTATAAGTTATGGGTAAGAAAGTTAAGAAGGCCGTGAAGAAAGTCACCAAGTCCGTTAAGAAAGTCGTTAAGGAAGGGGCTCGTCCGGTTAAACAGGTTGCTGGCGGTCTAGCTGGTCTGGCTGGTGGTACTGGTGAAGCACAGATGGTGGAAGTACCACAAGCTGCCGCACAGATTGTTGACGTACCTGAGAAAGAGGTTTCCACTGAGGACGAAGCacagac                                                                                                                                                                              <  1:337493/301‑1 (MQ=255)`  
                                                                                                                                      `ccATGATTGCGATTGGGTGTCTCGTTGTAAGCATGAAGGCGAGACAATGCGTGTATACTTTAAGGATGTATAAGTTATGGGTAAGAAAGTTAAGAAGGCCGTGAAGAAAGTCACCAAGTCCGTTAAGAAAGTCGTTAAGGAAGGGGCTCGTCCGGTTAAACAGGTTGCTGGCGGTCTggctggtggtactggtgaagcacagatggtgtaagtaccacaagctgccgcacagattgttgacgtacctgagaaagaggtttccactgaggacgaagcacagacagaaagcggacgcaagaaa                                                                                                                                                                    >  1:202154‑M1/1‑177 (MQ=255)`  
                                                                                                                                                        `gtCTCGTTGTAAGCATGAAGGCGAGACAATGCGTGTATACTTTAAGGAGGTATAAGTTATGGGTAAGAAAGTTAAGAAGGCCGTGAAGAAAGTCACCAAGTCCGTTAAGAAAGTCGTTAAGGAAGGTGCTCGTCCGGTTAAACAGGTTTCTGGCGGTCTAGCTGGTCTGGCTGGTGGTACTGGTGAAGCACAGATGGTGGAAGTACCACAAGCTGCCGCACAGATTATTGACGTACCTGAGAAAGAGGTTTCCACTGAGGACGAAGCACAGACAGCAAGCGGACGCAAGAAAGCTCGTGCt                                                                                                                                                  >  1:213645/1‑301 (MQ=255)`  
                                                                                                                                                             `ggctcggaaatgggtataagggacagcgtgtatactttaaggaggtataagTTATGGGTAAGAAAGTTAAGAAGGCCGTGAAGAAAGTCACCAAGTCAGTTAAGAAAGTCGTTAAGGAAGGGGCTCGTCCGGTTAAACAGGTTGCTGGCGGTCTggctggtggtactggtgaagcacagatggtggaagtaccacaagctgccgcacagattgttgacgtacctgagaaagaggtttccactgaggacgaagcacagacagaaagcggacgcaagaaagctcgtgctggcggtaagaaatc                                                                                                                                             <  1:189682‑M1/276‑148 (MQ=255)`  
                                                                                                                                                              `ttGTAAGCATGAAGGCGAGACAATGCGTGTATACTTTAAGGAGGTATAAGTTATGGGTAAGAAAGTTAAGAAGGCCGTGAAGAAAGTCACCAAGTCCGTTAAGAAAGTCGTTAAGGAAGGGGCTCGTCCGGTTAAACAGGTTGCTGGCGGTCTggctggtggtactggtgaagcacagatggtggaagtaccacaagctgccgcacagattgttgacgtacctgagaaagaggtttccactgaggacgaagcacagacagaaagcggccgcaagaaagctcgtgctggcggtaggaaatcc                                                                                                                                            >  1:262661‑M1/1‑153 (MQ=255)`  
                                                                                                                                                                `gTAAGCATGAAGGCGAGACAATGCGTGTATACTTTAAGTAGGTATAAGTTATGGGTAAGAAAGTTAAGAAGGCCGTGAAGAAAGTCACCAAGTCCGTTAAGAAAGTCGTTAAGGAAGGGGCTCGTCCGGTTAAACAGGTTGCTGGCGGTCTggctggtggtactggtgaagcacagatggtggaagtaccacaagctgccgcacagattgttgacgtacctgagaaagaggtttccactgaggacgaagcacagacagaaagcggacgcaagaaagatcgagttggcggtaagaaatcttt                                                                                                                                          >  1:145650‑M1/1‑151 (MQ=255)`  
                                                                                                                                                                   `gagaatgagtataagagaaaggtgtatactttaaggaggtataagtTATGGGGAAGAAAGTTAAGAAGGCCGTGAAGAAAGTCAACAAGTCCGTTAAGAAAGTCGTTAAGGAAGGGGCTCGTCCGGTTAAACAGGTTGCTGGCGGTCTggctggtggtactggtgaagcacagatggtggaagtaccacaagctgccgcaaagattgttgacgtacctgagaaagaggtttccactgaggacgaagcacagacagaaagcggacgcaagaaagctcgtgctggcggtaagaaatccttgag                                                                                                                                       <  1:281034‑M1/280‑154 (MQ=255)`  
                                                                                                                                                                    `gCATGAAGGCGAGACAATGCGTGTATACTTTAAGGAGGTATAAGTTATGGGTAAGAAAGTTAAGAAGGCCGTGAAGAAAGTCACCAAGTCCGTTAAGAAAGTCGTTAAGGAAGGGGCTCGTCCGGTTAAACAGGTTGCTGGCGGTCTggctggtggtactggtgaagcacagatggtggaagtaccacaagctgccgcacagattgttgacgtacctgagaaagaggtttccactgaggacgaagcacagacagaaagcggacgcaagaaagctcgtgctggcggtaagaaatcctctgtc                                                                                                                                      >  1:274352‑M1/1‑147 (MQ=255)`  
                                                                                                                                                                       `atgaGGGGAGACAAGGCGGGGAGACTTTAAGGAGGTATAAGGTATGGGTAAGAAAGTTAAGAAGGCCGTGAAGAAAGGCACCAAGTCCGTTAAGAAAGTCGTTAAGGAAGGGGCTCGTCCGGTTAAACAGGTTTATGGCGGTCTAGCTGGTCTGGCTGGTGGTACTGGTGAAGCACAGATGGTGGAAGTACCACAAGCTGCCGCACAGATTGTTGACGTACCTGAGAAAGAGGTTTCCACTGAGGACGAAGCACAGACAGAAAGCGGACGCAAGAAAGCTCGTGCTGGCGGTAAGAAATcc                                                                                                                                   <  1:126509/298‑1 (MQ=255)`  
                                                                                                                                                                        `gAAGGCGAGACAATGCGTGTATACTTTAAGGAGGTATAAGTGATGGGTAAGAAAGTTAAGAAGGCCGTGAAGAAAGTCACCAAGTCCGTTAAGAAAGTCGGTAAGGAAGGGGCTCGTCCGGTTAAACAGGTTGCTGGCGGTCTggctggtggtactggtgaagcacagatggtggaagtaccacaagctgccgcacagattgttgacgtacctgagaaagaggtttccactgaggacgaagcacagacagaaagcggacgcaagaaagctcgtgctggcggtaataaatccttgagtgtag                                                                                                                                  <  1:15705‑M1/301‑159 (MQ=255)`  
                                                                                                                                                                          `aGGCGAGACAATGCGTGTATACTTTAAGGAGGTATAAGTTATGGGTAAGAAAGTTAAGAAGGCCGTGAAGAAAGTCACCAAGTACGTTAAGAAAGTCGTTAAGGAAGGGGCTCGTCCGGTTAAACAGGTTTCTGGCGGTCTAGCTGGTCTGGCTGGTGGTACTGGTGAAGCACAGATGGTGGAAGTACCACAAGCTGCCGCACAGATTGTTGACGTACCTGAGAAAGAGGTTACCACTGAGGACGAAGCACAGACAGAAAGCGGACGCAAGAAAGCTCGTGCTGGCGGTAAGAAATCCTTg                                                                                                                                >  1:107342/1‑301 (MQ=255)`  
                                                                                                                                                                           `ggCGAGACAATGCGTGTATACTTTAATGAGGTATAAGTTATGGGTAAGAAAGTTAAGAAGGCCGTGAAGAAAGTCACCAAGTCCGTTAAGAAAGTCGTTAAGGAAGGGGCTCGTCCGGTTAAACAGGTTGCTGGCGGTCTagctggtggtactggtgaagcacagatggtggaagtaccacaagctgccgcacagattgtcgacgtacctgagaaagaggtttccactgaggacgaagcacagacagaaagcggaagcaagaaagctcgttctggcggtaagaaatccttgagtgtagccc                                                                                                                               >  1:3539‑M1/1‑140 (MQ=255)`  
                                                                                                                                                                                 `aaaatgCGTGTATACTTTAAGGAGGTATAAGTTATGGGTAAGAAAGTTAAGAAGGCCGTGAAGAAAGTCACCAAGTCCGTTAAGAAAGTCGTTAAGGAAGGGGCTCGTCCGGTTAAACAGGTTGCTGGCGGTCTggctggtggtactggtgaagcacagatggtggaagtaccacaagctgccgcacagattgttgacgtacctgagaaagaggtttccactgaggacgaagcacagacagaaagcggacgcaagaaagctcgtgctggcggtaagaaatccttgagtgtagcccgtagct                                                                                                                         <  1:192433‑M1/299‑168 (MQ=255)`  
                                                                                                                                                                                      `gCGTGTATACTTTAAGGAGGTATAAGTTATGGGTAAGAAAGTTGAGAAGGCCGTGAAGAAAGTCACCAAGTCCGTTAAGAAAGTCGTTAAGGAAGGGGCTCGTCCGGTTAAACAGGTTGCTGGCGGTCTggctggtggtactggtgaagcacagatggtggaagtaccacaagctgccgcacagattgttgacgtacctgagaaagaggtttccactgaggacgaagcacagacagaaagcggacgcaagaaagctcgtgctggcggtaagaaatccttgagtgtagcccgtagctccggt                                                                                                                    <  1:116586‑M1/301‑173 (MQ=255)`  
                                                                                                                                                                                      `gCGTGTATACTTTAAGGAGGTATAAGTTATGGGTAAGAAAGTTAAGAAGGCCGTGAAGAAAGTCACCAAGTCCGTTAAGAAAGTCGTTAAGGAATGGGCTCGTCCGGTTAAACAGGTTTCTGGCGGTCTAGCTGGTCTGGCTGGTGGTACTGGTGAAGCACAGATGGTGGAAGTACCACAAGCTGCCGCACAGATTGTTGACGTACCTGAGAAAGAGGTTTCCACTGAGGACGAAGCACAGACAGAAAGCGGACGCAAGAAAGCTCGTGCTGGCGGTAAGAAATCCTTGAgctgtctctta                                                                                                                    >  1:247947/1‑291 (MQ=255)`  
                                                                                                                                                                                      `gCGTGTATACTTTAAGGAGGTATAAGTTATGGGTAAGAAAGTTAAGAAGGCCGTGAAGAAAGTCACCAAGTCCGTTAAGAAAGTCGTTAAGGAAGGGGCTCGTCCGGTTAAACAGGTTTCTGGCGGTCTAGCTGGTCTGGCTGGTGGTACTGGTGAAGCACAGATGGTGGAAGTACCACAAGCTGCCGCACAGATTGTTGACGTACCTGAGAAAGAGGTTTCCACTGAGGACGAAGCACAGACAGAAAGCGGACGCAAGAAAGCTCGTGCTGGCGGTAAGAAATCCTTGAGTGTAGCCCGt                                                                                                                    >  1:138035/1‑301 (MQ=255)`  
                                                                                                                                                                                       `ggagtgtataagagaaaggtataagttatgggtaaGAAAGTTAAGAAGGCCGTGAAGAAAGTCACCAAGTCCGTTAAGAAAGTCGTTAAGGAAGGGGCTCGTCCGGTTAAACAGGTTGCTGGCGGTCTggctggtggtactggtgaagcacagatggtggaagtaccacaagctgccgcacagattgttgacgtacctgagaaagaggtttccactgaggacgaagcacagacagaaagcggacgcaagaaagctcgtgctggcggtaagaaatccttgagtgtagcccgtagctccggtg                                                                                                                   <  1:1960‑M1/285‑174 (MQ=255)`  
                                                                                                                                                                                       `cGTGTATACTTTAAGGAGGTATAAGTTATGGGTAAGAAAGTTAAGAAGGCCGTGAAGAAAGTCACCAAGTCCGTTAAGAAAGTCGTTAAGGAAGGGGCTCGTCCGGTTAAACAGGTTGCTGGCGGTCTAGCTGGTCTGGCTGGTGGTACTGGTGAAGCACAGATGGTGGAAGTACCACAAGCTGCCGCACAGATTGTTGACGTACCTGATAAAGAGGTTTCCACTGAGGACGAAGCACAGACAGAAAGCGGACGCAAGAAAGCTCGTGCTGGCGGTAAGAAATCCTTGAGTGTAGCCCGTa                                                                                                                   >  1:4783/1‑301 (MQ=255)`  
                                                                                                                                                                                               `cTTTAAGGAGGTATAAGTTATGGGTAAGAAAGTTAAGAAGGCCGTGAAGAAAGTCACCAAGTCCGTTAAGAAAGTCGTTAAGGAAGGGGCTCGTCCGGTTAAACAGGTTGCTGGCGGTCTggctggtggtactggtgaagcacagatggtggaagtaccacaagctgccgcacagattgttgacgtacctgagaaagaggtttccactgaggacgaagcacagacagaaagcggacgcaagaaagctcgtgctggcggtaagaaatccttgagtgtagcccgtagctccggtggcggtatc                                                                                                           >  1:329404‑M1/1‑120 (MQ=255)`  
                                                                                                                                                                                               `cTTTAAGGAGGTATAAGTTATGGGTAAGAAAGTTAAGAAGGCCGTGAAGAAAGTCACCAAGTCCGTTAAGAAAGTCGTTAAGGAAGGGGCTCGTCCGGTTAAACAGGTTGCTGGCGGTCTggctggtggtactggtgaagcacagatggtggaagtaccacaagctgccgcacagattgttgacgtacctgagaaagaggtttccactgaggacgaagcacagacagaaagcggacgcaagaaagctcgtgctggcggtaagaaatccttgagtgtagcccgtagctccggtggcggtatc                                                                                                           >  1:309920‑M1/1‑120 (MQ=255)`  
                                                                                                                                                                                                       `aggTATAAGTTATGGGTAAGAAAGTTAAGAAGGCCGTGAAGAAAGTCACCAAGTCCGTTAAGAAAGTCGTTAAGGAAGGGGCTCGTCCGGTTAAACAGGTTGCTGGCGGTCTggctggtggtactggtgaagcacagatggtggaagtaccacaagctgccgcacagattgttgacgtacctgagaaagaggtttccactgaggacgaagcacagacagaaagcggacgcaagaaagctcgtgctggcggtaagaaatccttgagtgtagcccgtagctccggtggcggtatcaacattta                                                                                                   >  1:166194‑M1/1‑112 (MQ=255)`  
                                                                                                                                                                                                       `aggTATAAGTTATGGGTAAGAAAGTTAAGAAGGCCGTGAAGAAAGTAACCAAGTCCGTTAAGAAAGTCGTTAAGGAAGGGGCTCGTCCGGTTAAACAGGTTGCTGGCGGTCTGGCTGGTCTGGCTGGTGGTACTGGTGAAGCACAGATGGTGGAAGTACCACAAGCTGCCGCACAGATTGTTGACGTACCTGAGAAAGAGGTTTCCACTGAGGACGAAGCACAGACAGAAAGAGGACGCAAGAAAGCTCGTGCTGGCGGTAAGAAATCCTTGAGTGTAGCCCGTAGCTCCGGTGGCGGTAt                                                                                                   >  1:312296/1‑301 (MQ=255)`  
                                                                                                                                                                                                        `ggTATAAGTTATGGGTAAGATAGTTAAAAAGGCCGTGAAGAAAGTCACCAAGTCCGTTAAGAAAGTCGTTAAGGAAGGGGCTCGTCCGGTTAAACAGGTTTCTGGCGGTCTAGCTGGTCTGGCTGGTGGTACTGGTGAAGCACAGATGGTGGAAGTACCACAAGCTGCCGCACAGATTGTTGACGTACCTGAGAAAGAGGTTTCCACTGAGGACGAAGCACAGACAGAAAGCGGACGCAAGAAAGCTCGTGCTGGCGGTAAGAAATCCTTGAGTGTAGCCCGTAGCTCCGGTGGCGGTATc                                                                                                  >  1:265491/1‑301 (MQ=255)`  
                                                                                                                                                                                                        `ggTATAAGTTATGGGTAAGAAAGTTAAGAAGGCCGTGAAGAAAGTCACCAAGTCCGTTAAGAAAGTCGTTAAGGAAGGGGCTCGTCCGGTTAAACAGGTTTCTGGCGGTCTAGCTGGTCTGGCTGGTGGTACTGGTGAAGCACAGATGGTGGAAGTACCACAAGCTGCCGCACAGATTGTTGACGTACCTGAGAAAGAGGTTTCCACTGAGGACGAAGCACAGACAGAAAGCGGACGCAAGAAAGCTCGTGCTGGCGGTAAGAAATCCTTGAGTGTAGCCCGTAGCTCCGGTGGCGGTATc                                                                                                  >  1:105281/1‑301 (MQ=255)`  
                                                                                                                                                                                                        `ggTATAAGTTATGGGTAAGAAAGTTAAGAAGGCCGTGAAGAAAGTCACCAAGTCCGTTAAGAAAGTCGTTAAGGAAGGGGCTCGTCCGGTTAAACAGGTTGCTGGCGGTCTggctggtggtactggtgaagcacagatggtggaagtaccacaagctgccgcacagattgttgacgtacctgagaaagaggtttccactgaggacgaagcacagacagaaagcggacgcaagaaagctcgtgctggcggtaagaaatccttgagtgtagcccgtagctccggtggcggtatcaacatttaa                                                                                                  >  1:219998‑M1/1‑111 (MQ=255)`  
                                                                                                                                                                                                         `gTATAAGTTATGGGTAAGAAAGTTAAGAAGGCCGTGAAGAAAGTCACCAATTCCGTTAAGAAAGTCGTTAAGTAAGGGGCTCGTCCGGTTAAACAGGTTGCTGGCGGTCTggctggtggtactggtgacgcacagatggtggaagtaccacaagctgccgcacagattgttgacgtacctgagaaagaggtttccactgaggacgaagcacagacagaaagcggacgcaagaaagctcgtgctggcggtaagaaatccttgagtgtagcccgtagctccggtggcggtatcaacatttaat                                                                                                 >  1:110867‑M1/1‑110 (MQ=255)`  
                                                                                                                                                                                                         `gTATAAGTTATGGGTAAGAAAGTTAAGAAGGCCGTGAAGAAAGTCACCAAGTCCGTTAAGAAAGTCGTTAAGGAAGGGGCTCGTCCGGTTAAACAGGTTTCTGGCGGTCTAGCTGGTCTGGCTGGTGGTACTGGTGAAGCACAGATGGTGGAAGTACCACAAGCTGCCGCACAGATTGTTGACGTACCTGAGGAAGAGGTTTCCACTGAGGACGAAGCACAGACAGAAAGCGGACGCAAGAAAGCTCGTGCTGGCGGTAAGAAATCCTTGAGTGTAGCCCGTAGCTCCGGTGGCAGTATct                                                                                                 >  1:242828/1‑300 (MQ=255)`  
                                                                                                                                                                                                         `gTATAAGTTATGGGTAAGAAAGTTAAGAAGGCCGTGAAGAAAGTCACCAAGTCCGTTAAGAAAGTCGTTAAGGAAGGGGCTCGTCCGGTTAAACAGGTTTCTGGCGGTCTAGCTGGTCTGGCTGGTGGTACTGGTGAAGCACAGATGGTGGAAGTACCACAAGCTGCCGCACAGATTGTTGACGTACCTGAGAAAGAGGTTTCCACTGAGGACGAAGCACAGACAGAAAGCGGACGCAAGAAAGCTCGTGCTGGCGGTAAGAAATCCTTGAGTGTAGCCCGTAGCTCCGGTGGCGGTATCa                                                                                                 >  1:291464/1‑301 (MQ=255)`  
                                                                                                                                                                                                         `gTATAAGTTATGGGTAAGAAAGTTAAGAAGGCCGTGAAGAAAGTCACCAAGTCCGTTAAGAAAGTCGTTAAGGAAGGGGCTCGTCCGGTTAAACAGGTTGCTGGCGGTCTggctggtggtactggtgaagcacagatagtggaagtaccacaagctgccgcacagattgttgacgtacctgagaaagaggtttccactgaggacgaagcacagacagaaagcggacgcaagaaagctcgtgctggcggtaagaaatccttgagtgtagcccgtagctccggtggcggtatcaacactgtct                                                                                                 >  1:140644‑M1/1‑110 (MQ=255)`  
                                                                                                                                                                                                          `tataAGTTGTGGGTAAGTAAATCAAGAAGGCCGTGAAGAAAGTCACCAAGTCCGTTAAGAAAGTCGTTAAGGAAGGGGATCGTGCGGTTAGACAGGTTGCTGGCGGTCTggctggttgtactggtgaagcacagatggtggaagtaccacaagctgccgcacagattgttgacgtacctgagaaagaggtttccactgaggacgaagcacagacagaaagcggacgcaagaaagctcgtgctggcggtaagaaatccttgagtgtagcccgtagctccggtggcggtatcaacatttaatc                                                                                                <  1:287202‑M1/301‑193 (MQ=255)`  
                                                                                                                                                                                                          `tataAGTTATGGGTAAGAAAGTTAAGAAGGCCGTGAAGAAAGTCACCAAGTCCGTTAAGAAAGTCGTTAAGGAAGGGGCTCGTCCTGTTAAACAGGTTGCTGGCGGTCTggctggtggtactggtgaagcacagatggtggaagtaccacaagctgccgcacagattgttgacgtacctgagaaagaggtttccactgaggacgaagcacagacagaaagcggacgcaagaaagctcgtgctggcggtaagaaatccttgagtgtagcccgtagctccggtggcggtatcaacatttaatc                                                                                                <  1:57216‑M1/301‑193 (MQ=255)`  
                                                                                                                                                                                                               `gTTATGGGTAAGAAAGTTAAGAAGGCCGTGAAGAAAGTCACCAAGTCCGTTAAGAAAGTCGTTACGGAAGTGGCTCGTCCGGTTAAACAGGTTGCTGGCGGTTTggctggtggtactggtgaagcacagatggtggaagtaccacaagctgccgcacagattgttgacgtacctgagaacgaggtttccactgaggacgaagcacagacagaacgcggccgcaaacaagctcgtgctggcggtaagaaatccttgagtgtagcacgttactccggtggcggtatccacatttactcaggag                                                                                           >  1:452‑M1/1‑104 (MQ=255)`  
                                                                                                                                                                                                                            `gctcggagatgtgtataagagacagcaccaagtccgttaagcaagtcgttaAGGAAGGGGCTCGTCCGGTTAAACAGGTTGCTGGCGGTCTggctggtggtactggtgaagcacagatggtggaagtaccacaagctgccgcacagattgttgacgtacctgagaaagaggtttccactgaggacgaagcacagacagaaagcgtacgcaagaaagctcgtgctggcggtaagaaatccttgagtgtagcccgtagctccggtgtcggtatcaacatttaatcaggaggttatcgtggaag                                                                              <  1:77019‑M1/276‑211 (MQ=255)`  
                                                                                                                                                                                                                             `aGTTAAGAAGGCCGTGAAGAAAGTCACCAAGTCCGTTAAGAAAGTCGTTAAGGAAGGGGCTCGTCCGGTTAAACAGGTTGCTGGCGGTCTggctggtggtactggtgaagcacagatggtggaagtaccacaagctgccgcacagattgttgacgtacctgagaaagaggtttccactgaggacgaagcacagacagaaagcggacgcaagaaagctcgtgctggcggtaagaaatccttgagtgtagcccgtagctccggtggcggtatcaacatttaatcaggaggttatcgtgctgtc                                                                             >  1:133393‑M1/1‑90 (MQ=255)`  
                                                                                                                                                                                                                              `gTTAAGAAGGCCGTGAAGAAAGTCACCAAGTCCGTTAAGAAAGTCGTTAAGGAAGGGGCTAGTCAGGTTAAACAGGTTGCTGGCGATCTAGCTGGTCTGGCTGGTGGTACTGGTGAAGCACAGATGGTGGAAGTACCACAAGCTGCCGCACAGATTGTTGACGTACCTGAGAAAGAGGTTTCCACTGAGGACGAAGCACAGACAGAAAGCGGACGCAAGAAAGCTCGTGCTGGCGGTAAGAAATCCTTGAGTGTAGCCCGTAGCTCCGGTGGCGGTATCAACATTTAATcctgtctcttat                                                                            >  1:73933/1‑290 (MQ=255)`  
                                                                                                                                                                                                                                          `gTGAAGAAAGTCACCAAGTCCGTTAAGAAAGTCGTTAAGGAAGGGGCTCGTCCGGTTAAACAGGTTGCTGGCGGTCTtgctggtggtactggtgaagcacagatggtggaagtaccacaagctgccgcacagattgttgacgtacctgagaaagaggtttccactgaggacgaagcacagacagaaagcggacgcaagaaagctcgtgctggcggtaagaaatccttgagtgtagcccgtagctccggtggcggtatcaacatttaatcaggaggttatcgtggaagactgcattgaatgg                                                                >  1:257407‑M1/1‑77 (MQ=255)`  
                                                                                                                                                                                                                                                     `cACCAAGTCCGTTAAGAAAGTCGTTAAGGAAGGGGCTCGTCCGGTTAAACAGGTTGCTGGCGGTCTAGCTGGTCTGGCTGGTGGTACTGGTGAAGCACAGATGGTGGAAGTACCACAAGCTGCCGCACAGATTGTTGACGTACCTGAGAAAGAGGTTTCCACTGAGGACGAAGCACAGACAGAAAGCGGACGCAAGAAAGCTCGTGCTGGCTGTAAGAAATCCTTGAGTGTAGCCCGTAGCTCCGGTGGCGGTATCAACATTTAATCAGGAGGTTATCGTGGAAGACTGCATTGAATGTAc                                                     >  1:148868/1‑301 (MQ=255)`  
                                                                                                                                                                                                                                                         `ggggctcggagaagaggaaaagagaaaggggctcgtcctgttaaacaggttgctGGCGGCCTagctgggggtactggtgaagaacagatggtggaagtaccacaagctgccgcacagattgttgacgtacctgagaaagaggtttccactgaggacgaagcacagacagaaagcggacgcaagaaagctcgtgctggcggtaagaaatccttgagtgtagcccgtagctccggtggcggtatcaacttttaatcaggaggttatcgtggaagactgcattgaatggaccggaggtgtcaac                                                 <  1:160678‑M1/276‑240 (MQ=255)`  
                                                                                                                                                                                                                                                           `gTCCGTTAAGAAAGTCGTTAAGGAAGGGGCTCGTCCGGTTAAACAGGTTGCTGGCGGTCTggctggtggtactggtgaagcacagatggtggaagtaccacaagctgccgcacagattgttgacgtacctgagaaagaggtttccactgaggacgaagcacagacagaaagcggacgcaagaaagctcgtgctggcggtaagaaatccttgagtgtagcccgtagctccagtggcggtatcaacatttaatcaggaggttatcgtggaagactgcattgaatggaccggaggtgtcaactc                                               >  1:29550‑M1/1‑60 (MQ=255)`  
                                                                                                                                                                                                                                                                 `tAAGAAAGTCGTTAAGGAAGGGGCTCGTCCGGTTAAACAGGTTGCTGGCGGTCTggctggtggtactggtgaagcacagatggtggaagtaccacaagctgccgcacagattgttgacgtacctgagaaagaggtttccactgaggacgaagcacagacagaaagcggacgcaagaaagctcgtgctggcggtaagaaatccttgagtgtagcccgtagctccggtggcggtatcaacatttaatcaggaggttatcgtggaagactgcattgaatggaccggaggtgtcaactctaaggg                                         <  1:171181‑M1/301‑248 (MQ=255)`  
                                                                                                                                                                                                                                                                   `aGAAAGTCGTTAAGGAAGGGGCTCGTCCGGTTAAACAGGTTTCTGGCGGTCTAGCTGGTCTGGCTGGTGGTACTGGTGAAGCACAGATGGTGGAAGTACCACAAGCTGCCGAACAGATTGTTGACGTACCTGAGAAAGAGGTTTCCACTGAGGACGAAGCACAGACAGAAAGCGGACGCAAGAAAGCTCGTGCTGGCGGTAAGAAATCCTTGATTGTAGCCCGTAGCTCCGGTGGCGGTATCAACATTTAATCAGGAGGTTATCGTGGAAGACTGCATTGAATGGACCGGAGGTGTCAAct                                       <  1:323284/301‑1 (MQ=255)`  
                                                                                                                                                                                                                                                                   `aGAAAGTCGTTAAGGAAGGGGCTCGTCCGGTTAAACAGGTTGCTGGCGGTCTggctggtggtactggtgaagcacagatggtggaagtaccacaagctgccgcacagattgttgacgtacctgagaaagaggtttccactgaggacgaagcacagacagaaagcggacgcaagaaagctcgtgctggcggtaagaaatccttgagtgtagcccgtagctccggtggcggtatcaacatttaatcaggaggttatcgtggaagactgcattgaatggaccggaggtgtcaactctaagggtt                                       >  1:113625‑M1/1‑52 (MQ=255)`  
                                                                                                                                                                                                                                                                    `gAAAGTCGTTAAGGAAGGGGCTCGTCCGGTTAAACAGGTTGCTGGCGGTCTggctggtggtactggtgaagcacagatggtggaagtaccacaagctgccgcacagattgttgacgtacctgagaaagaggtttccactgaggacgaagcacagacagaaagcggacgcaagaaagctcgtgctggcggtaagaaatccttgagtgtagcccgtagctccggtggcggtatcaacatttaatcaggaggttatagtggaagactgcattgaatggacaggaggtgtcaactctaagggtta                                      >  1:177249‑M1/1‑51 (MQ=255)`  
                                                                                                                                                                                                                                                                        `ggcggtaagggAGGGGCTCGTCCGGTTAAACAGGTTGCTGGCGGTCTggctggtggtactggtgaagcacagatggtggaagtaccacaagctgccgcacagattgttgacgtacctgagaaagaggtttccactgaggacgaagcacagacagaaagcggacgcaagaaagctcgtgctggcggtaagaaatccttgagtgtagcccgtagctccggtggcggtatcaacatttaatcaggaggttatcgtggaagactgcattgaatggaccggaggtgtcaactctaagggttatggt                                  <  1:217563‑M1/296‑255 (MQ=255)`  
                                                                                                                                                                                                                                                                        `gTCGTTAAGGAAGGGGCTCGTCCGGTTAAACAGGTTGCTGGCGGTCTggctggtggtactggtgaagcacagatggtggaagtaccacaagctgccgcacagattgttgacgtacctgagaaagaggtttccactgaggacgaagcacagacagaaagcggacgcaagaaagctcgtgctggcggtaagaaatccttgagtgtagcccgtagctccggtggcggtatcaacatttaataaggaggttatcgtggaagactgcattgaatggaccggaggtgtcaactctaagggttatggt                                  <  1:250862‑M1/301‑255 (MQ=255)`  
                                                                                                                                                                                                                                                                        `gTCGTTAAGGAAGGGACTCGTCCGGTTAAACAGGTTGCTGGCGGTCTggctggtggtactggtgaagcacagatggtggaagtaccacaagctgccgcacagattgttgacgtacctgagaaagaggtttccactgaggacgaagtacagacagaaagcggacgcaagaaagctcgtgctggcggtaagaaatccttgagtgtagcccgtagctccggtggcggtatcaacatttaatcaggaggttatcgtggaagactgcattgaatggaccggaggtgtcaactctaagggttatggt                                  <  1:222087‑M1/301‑255 (MQ=255)`  
                                                                                                                                                                                                                                                                              `tgtgtataagagacagcggttaaacaggttgctGGCGGTCTggctggtggtactggtgaagcacagatggttgaagtaccacaagctgccgcacagattgttgacgtacctgagaaagaggtttccactgaggacgaagcacagacagaaagcggacgcaagaaagctcgtgctggcggtaagaaatccttgagtgtagcccgtagctccggtggcggtatcaacatttaatcaggaggttatcgtggaagactgcattgaattgaccggaggtgtcaactctaagggttatggtcgtaag                            <  1:64108‑M1/285‑261 (MQ=255)`  
                                                                                                                                                                                                                                                                              `agggaaggggCTGGCACGGTTAATAAGGTTGCTGGAGGTCTggctggtggtactgtggaagcacagatggtggaagtaccacaagctgccgaacagattgttgactgacctgagaaagaggtttccactgaggacgaagaacagacagaaagcggacgcaagaaagttcgtgctggcggtaagaaatccttgagtgtagcccgtagctccggtggcggtatcaacatttaatcaggaggttagcgtggaagactgcattgaatggaccggaggtgtcaactctaagggttatggtcgtaag                            <  1:319949‑M1/299‑261 (MQ=255)`  
                                                                                                                                                                                                                                                                              `aaggaaggGGCTCGTCCGGTTAAACAGGTTGCTGGCGGTCTggctggtggtactggtgaagcacagatggtggaagtaccacaagctgccgcacagattgttgacgtacctgagaaagaggtttccactgaggacgaagcacagacagaaagcggacgcaagaaagctcgtgctggcggtaagaaatccttgagtgtagccagtagctccggtggcggtatcaacatttaaacaggaggttatcgtggaagactgcattgaatggaccggaggtgtcaactctaagggttatggtcgtaag                            <  1:85898‑M1/301‑261 (MQ=255)`  
                                                                                                                                                                                                                                                                               `aggaaggGGCTCGTCCGGTTAAACAGGTTGCTGGCGGTCTggctggtggtactggtgaagcacagatggtggaagtaccacaagctgccgcacagattgttgacgtacctgagaaagaggtttccactgaggacgaagcacagacagaaagcggacgcaagaaagctcgtgctggcggtaagaaatccttgagtgtagcccgtagctccggtggcggtatcaacatttaatcaggaggttatcgtggaagactgcattgaatggaccggaggtgtcaactctaagggttatggtcgtaagt                           <  1:245586‑M1/301‑262 (MQ=255)`  
                                                                                                                                                                                                                                                                                         `tCGTCCGGTTAAACAGGTTGCTGGCGGTCTgggtggtggtactggtgaagcacagatggtggaagtaccacaagctgccgcacagattgttgacgtacctgagaaagaggtttccactgaggacgaagcacagacagaaagcggacgcaagaaagctcgtgctggcggtaagaaatccttgagtgtagcccgtagctccggtggcggtatcaacatttaatcaggaggttatcgtggaagactgcattgaatggaccggaggtgtcaactctaagggttatggtcgtaagtgggttaatgg                 <  1:82873‑M1/301‑272 (MQ=255)`  
                                                                                                                                                                                                                                                                                          `cGTCCGGTTAAACAGGTTGCTGGCGGTCTggctggtggtactggtgaagcacagatggtggaagtaccacaagctgccgcacagattgttgacgtacctgagaaagaggtttccactgaggacgaagcacagacagaaagcggacgcaagaaagctcgtgctggcggtaagaaatccttgagtgtagcccgtagctccggtggcggtatcaacatttaatcaggaggttatcgtagaagactgcattgaatggaccggaggtgtcaactctaagggttatggtcgtaagtgggttaatggt                <  1:209331‑M1/301‑273 (MQ=255)`  
                                                                                                                                                                                                                                                                                              `cGGTTAAACAGGTTGCTGGCGGTCTagctggcggtactggtgaagcacagatggtggaagtaccacaagctgccgcacagattgttgacgtacctgagaaagaggtttccactgaggacgaagcacagacagaaagcggacgcaagaaagctcgtgctggcggtaagaaatccttgagtgtagcccgtagctccggtggcggtatcaacatttaatcaggaggttatcgtggaagactgcattgaatggaccggaggtgtcaactctaagggttatggtcgtaagtgggttaatggtaaac            <  1:184860‑M1/301‑277 (MQ=255)`  
                                                                                                                                                                                                                                                                                                `gTTAAACAGGTTTCTGGCGGTCTAGCTGGTCTGGCTGGTGGTACTGGTGAAGCACAGATGGTGGAAGTACCACAAGCTGCCGCACAGATTGTTGACGTACCTGAGAAAGAGGTTTCCACTGAGGACGAAGCACAGACAGAAAGCGGACGCAAGAAAGCTCGTGCTGGCGGTAAGAAATCCTTGAGTGTAGCCCGTAGCTCCGGTGGCGGTATCAACATTTAATCAGGAGGTTATCGTGGAAGACTGCATTGAATGGACCGGAGGTGTCAACTCTACGGGTTATGGTCGTAAGTTGGTTAAt          >  1:61519/1‑301 (MQ=255)`  
                                                                                                                                                                                                                                                                                                `gTTAAACAGGTTGCTGGCGGTCTggctggtggtactggtaaagcacaggtggtggaagtaccacaagatgcggaacagattgttgacgtacctaagaaagaggtttccactgaggacaaagcacagacagaaagcggacgcaagaaagctcgtgctggcggtaagaaatccttgagtgtagcctgtagctccggtggcggtatcaacatttaatcaggaggttatcgtggaagactgcattgaatggaccggaggtgtcaactctaagggttatggtcgtaagtgggttaatggtaaactt          <  1:66863‑M1/301‑279 (MQ=255)`  
                                                                                                                                                                                                                                                                                                  `tAAACAGGTTGCTGGCGGTCTggctggtggtactggtgaagcacagatggtggaagtaccacaagctgccgcacagattgttgacgtaccttagaaagaggtttccactgaggacgaatcacagacatatctcggacgcaactaagctcgtgctggcggtatgaaatccttgtgtgtagcccgtagctccggtggcggtctcaacatttaatcaggaggttatcgtggaagactgcattgaatgtaccggaggtgtcaactctaagggttatggtcgtaagtgggttaatggtaacctttt        >  1:100102‑M1/1‑21 (MQ=255)`  
                                                                                                                                                                                                                                                                                                        `ggTTGCTGGCGGTCTggctggtggtactggtgaagcacagatggtggaagtaccacaagctgccgcacagattgttgacgtacctgagaaagaggtttccactgaggacgaagcacagacagaaagcggacgcaagaaagctcgtgctggcggtaagaaatccttgagtgtagcccgtagctccggtggcggtatcaacatttaatcaggaggttatcgtggaagactgcattgaatggaccgtaggtgtcaactctaagggttatggtcgtaagtgggttaatggtaaacttgtgactcc  >  1:239847‑M1/1‑15 (MQ=255)`  

                                                                                                                                                                                                                                                                                                            `|`                                                                                                                                                                                                                                                                                                          
`GCGTGGTGCGTAAGCTGTGACATTCACCACAAAGGAGACATTCTGGATGTAACGTCCGTTGTCATTAATCCTGAGGCAGACTCTAAGGGCTTACAGCGATTCCTAGCGAAACGCTTTAAGTACCTTGCGGAACTCCACGATTGCGATTGGGTGTCTCGTTGTAAGCATGAAGGCGAGACAATGCGTGTATACTTTAAGGAGGTATAAGTTATGGGTAAGAAAGTTAAGAAGGCCGTGAAGAAAGTCACCAAGTCCGTTAAGAAAGTCGTTAAGGAAGGGGCTCGTCCGGTTAAACAGGTTGCTGGCGGTCTAGCTGGTCTGGCTGGTGGTACTGGTGAAGCACAGATGGTGGAAGTACCACAAGCTGCCGCACAGATTGTTGACGTACCTGAGAAAGAGGTTTCCACTGAGGACGAAGCACAGACAGAAAGCGGACGCAAGAAAGCTCGTGCTGGCGGTAAGAAATCCTTGAGTGTAGCCCGTAGCTCCGGTGGCGGTATCAACATTTAATCAGGAGGTTATCGTGGAAGACTGCATTGAATGGACCGGAGGTGTCAACTCTAAGGGTTATGGTCGTAAGTGGGTTAATGGTAAACT  >  NC_001604.1/19325‑19921`

  

Alignment Legend

Aligned base mismatch/match (shaded by quality score): `ATCG/ATCG` `< 3 ≤` `ATCG/ATCG` `< 11 ≤` `ATCG/ATCG` `< 25 ≤` `ATCG/ATCG` `< 36 ≤` `ATCG/ATCG` `< 38 ≤` `ATCG/ATCG`

Unaligned base: `atcg`    Masked matching base: `atcg`    Alignment gap: `‑`    Deleted base: `‑`
